# The negative relationship between patients with NSCLC harbored STK11/KEAP1 copy number variation and immune microenvironment infiltration

**DOI:** 10.1186/s12967-021-02924-0

**Published:** 2021-06-14

**Authors:** Chenyue Zhang

**Affiliations:** 1 Department of Integrated Therapy, Fudan University Shanghai Cancer Center, Shanghai, 200032 China; 2 Department of Oncology, Shanghai Medical College, Fudan University, Shanghai, 200032 China

To the editor,

Studies have found STK11 mutation resistant to immune checkpoint inhibitors (ICIs) and worse survival in KEAP1 mutant patients compared with wildtype in response to ICIs [1, 2]. It has demonstrated that less immune cell infiltration could be found in patients with non-small cell lung cancer (NSCLC) harbored STK11/KEAP1 mutation, which maybe lead to the resistance or worse survival to immune checkpoint inhibitors (ICIs) [3, 4]. However, there have been no relevant studies investigating the association between STK11/KEAP1 copy number variation and immune microenvironment in patients with NSCLC. In this regard, we aim to interrogate the immune microenvironment in patients with NSCLC harboring STK11/KEAP1 copy number variation.

In the present study, we conducted an analysis by evaluating the immuno-contexture in patients with lung adenocarcinoma (LUAD) and lung squamous cell carcinoma (LUSC) harboring STK11/KEAP1 copy number variation using TIMER databases [5, 6]. We compared the tumor infiltration immune cells with different copy number variation including deep deletion, arm-level deletion, diploid/normal, arm-level gain, high amplification for KEAP1 and STK11 in LUAD and LUSC. The immune cells tested were primarily responsible for efficacious antitumor immunity, characterized by CD8 + T cell, CD4 + T cell and myeloid dendritic cell.

The results showed that for LUAD, arm level gain of STK11 was associated with debilitated immersion of CD8 + T cell (*P* = 0.0062). And arm level deletion of STK11 was correlated with CD4 + T cell infiltration (*P* = 0.0016). Both arm level deletion (*P* = 8.4e–06) and arm level gain (*P* = 0.0085) of STK11 was related with myeloid dendritic cells (Fig. 1A). For LUSC, the association could be found between STK11 arm level gain and CD8 + T cell (*P* = 0.034), between STK11 arm level deletion (*P* = 0.023) and myeloid dendritic cells, between STK11 arm level gain (*P* = 0.00033) and myeloid dendritic cells (Fig. 1B).

**Fig. 1:**
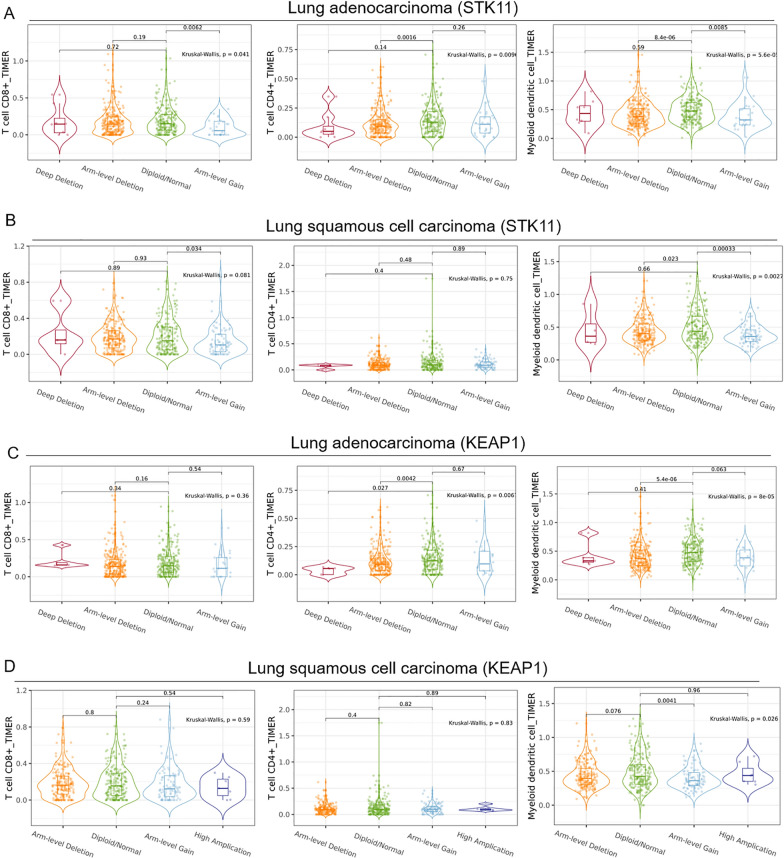
The association between patients with NSCLC harboring STK11/KEAP1 copy number variation and immune microenvironment infiltration. **A** The association between STK11 copy number alterations (deep deletion, arm-level deletion, diploid/normal, arm-level gain) and the infiltration of immune cells (CD8 + T cell, CD4 + T cells, myeloid dendritic cell) was analyzed in lung adenocarcinoma. **B** The association between STK11 copy number alterations (deep deletion, arm-level deletion, diploid/normal, arm-level gain) and the infiltration of immune cells (CD8 + T cell, CD4 + T cells, myeloid dendritic cell) was analyzed in lung squamous cell carcinoma. **C** The association between KEAP1 copy number alterations (deep deletion, arm-level deletion, diploid/normal, arm-level gain) and the infiltration of immune cells (CD8 + T cell, CD4 + T cells, myeloid dendritic cell) was analyzed in lung adenocarcinoma. **D** The association between KEAP1 copy number alterations (arm-level deletion, diploid/normal, arm-level gain, high amplification) and the infiltration of immune cells (CD8 + T cell, CD4 + T cells, myeloid dendritic cell) was analyzed in lung squamous cell carcinoma

As we further explored, the association of KEAP1 deep deletion (*P* = 0.027), arm level deletion (*P* = 0.0042) with CD4 + T cell infiltration was found in LUAD. And arm level deletion (*P* = 5.4e–06), arm level gain (*P* = 0.063) of KEAP1 were found to be linked with myeloid dendritic cells in LUAD (Fig. 1C). For LUSC, arm level gain of KEAP1 was associated with relatively less myeloid dendritic cell infiltration, as demonstrated in Fig. 1D.

In conclusion, for the first time, we have demonstrated the existence of dampened immune microenvironment in patients with NSCLC harboring STK11/KEAP1 copy number variation. However, it has to be noted that not all forms of copy number variation are linked with less immune cell immersion. Our study provides new insights into the immunological landscape of NSCLC harboring STK11/KEAP1 copy number variation, with relevance for therapeutic intervention. For patients with NSCLC harboring some forms of STK11/KEAP1 copy number variation, little immune cell infiltration is involved. Therefore, more complex treatment strategies may be needed to rekindle immune responses.

## Data Availability

The datasets generated during and/or analyzed during the current study are available from the corresponding author on reasonable request.

## References

[1] Skoulidis F, Goldberg ME, Greenawalt DM, Hellmann MD, Awad MM, Gainor JF (2018). STK11/LKB1 mutations and PD-1 inhibitor resistance in KRAS-mutant lung adenocarcinoma. Cancer Discov.

[2] Chen X, Su C, Ren S, Zhou C, Jiang T (2020). Pan-cancer analysis of KEAP1 mutations as biomarkers for immunotherapy outcomes. Ann Transl Med.

[3] Marinelli D, Mazzotta M, Scalera S, Terrenato I, Sperati F, D'Ambrosio L (2020). KEAP1-driven co-mutations in lung adenocarcinoma unresponsive to immunotherapy despite high tumor mutational burden. Ann Oncol.

[4] Wang H, Guo J, Shang X, Wang Z (2020). Less immune cell infiltration and worse prognosis after immunotherapy for patients with lung adenocarcinoma who harbored STK11 mutation. Int Immunopharmacol.

[5] Li T, Fan J, Wang B, Traugh N, Chen Q, Liu JS (2017). TIMER: a web server for comprehensive analysis of tumor-infiltrating immune cells. Cancer Res.

[6] Li B, Severson E, Pignon JC, Zhao H, Li T, Novak J (2016). Comprehensive analyses of tumor immunity: implications for cancer immunotherapy. Genome Biol.

